# Evaluation of the Potential Benefits of Trimetazidine in Metabolic Dysfunction-Associated Steatotic Liver Disease: A Randomized Controlled Trial

**DOI:** 10.3390/ph18091279

**Published:** 2025-08-27

**Authors:** Maha Youssif, Ragaey Ahmad Eid, Hoda Rabea, Yasmin M. Madney, Arwa Khaled, Khalid Orayj, Dina Attia, Engy A. Wahsh

**Affiliations:** 1 Department of Clinical Pharmacy, Faculty of Pharmacy, October 6 University, Giza 12585, Egypt; 2 Department of Gastroenterology, Hepatology, and Infectious Diseases (Tropical Medicine Department), Faculty of Medicine, Beni-Suef University, Beni-Suef 62514, Egypt; ragaeyahmad@med.bsu.edu.eg (R.A.E.); dina.attia@med.bsu.edu.eg (D.A.); 3 Department of Clinical Pharmacy, Faculty of Pharmacy, Beni-Suef University, Beni-Suef 62521, Egypt; hoda.ahmed@pharm.bsu.edu.eg (H.R.); yasminmohamed5050@yahoo.com (Y.M.M.); 4 Department of Clinical Pharmacy, College of Pharmacy, King Khalid University, Abha 62529, Saudi Arabia; arwak@kku.edu.sa (A.K.); korayj@kku.edu.sa (K.O.)

**Keywords:** MASLD, metabolic dysfunction-associated steatotic liver disease, trimetazidine (TMZ), CAP, inflammation, steatosis

## Abstract

**Background/Objectives:** Metabolic dysfunction-associated steatotic liver disease (MASLD) represents a significant global public health issue, affecting approximately 25% of the population and currently offering limited treatment options. Trimetazidine (TMZ) serves as a metabolic modulator that shifts cellular energy metabolism from fatty acid oxidation to glucose oxidation, thereby providing a novel therapeutic strategy aimed at addressing the underlying metabolic dysfunctions that contribute to the pathogenesis of MASLD. Our study aims to assess the efficacy of trimetazidine in improving hepatic steatosis, inflammation, and various metabolic parameters. **Methods:** In this double-masked, randomized controlled trial, 60 patients with confirmed MASLD diagnoses were randomly assigned in a 1:1 ratio to receive either trimetazidine 20 mg three times daily or a placebo, alongside lifestyle modifications, for 24 weeks. The trial was conducted in accordance with the Declaration of Helsinki and approved by the ethics committees of both participating institutions. We measured changes in hepatic steatosis, non-invasive fibrosis scores, inflammatory markers (including interleukin-6, tumor necrosis factor-alpha, and highly sensitive C-reactive protein), liver enzymes, and lipid profiles at baseline and at the end of the 24 weeks. **Results:** Hepatic steatosis decreased significantly, with controlled attenuation parameter scores from 352.5 to 302 dB/m in the TMZ group compared to the control (*p* < 0.001). TNF-α was reduced significantly in the TMZ group compared to the control group (*p* = 0.001). Fibrosis to AST score decreased from 0.49 to 0.25 in the TMZ group (*p* < 0.001). Aspartate aminotransferase decreased significantly compared to the control group (*p* 0.032). Notably, TMZ also imparted cardioprotective benefits, reducing total cholesterol by 14%, LDL by 17% (both *p* < 0.05), and triglycerides by 16% (*p* = 0.176). **Conclusions:** This groundbreaking trial supports the potential of trimetazidine as a promising therapeutic agent for MASLD, indicating substantial improvements in hepatic steatosis, inflammation, and metabolic disturbances. These findings underscore the urgency and importance of further multicenter trials to validate trimetazidine’s efficacy as a disease-modifying therapy for MASLD.

## 1. Introduction

Metabolic dysfunction-associated steatotic liver disease (MASLD) is the most common form of chronic liver disease globally [1]. Its defining features are the histologic manifestations of lobular inflammation, ballooning degeneration, and hepatic steatosis, with or without fibrosis [2]. Approximately a quarter or so of MASLD patients progress to steatohepatitis (MASH), which increases the risk of end-stage liver disease [3]. Metabolically associated steatohepatitis (MASH) is the subgroup of MASLD that can potentially lead to adverse clinical outcomes [4]. MASLD developed during hepatic steatosis (identified using non-invasive techniques and liver biopsy), which was present [5].

MASLD is a multifactorial, complex disease associated with metabolic disorders such as obesity, type 2 diabetes (T2DM), hypertension, and dyslipidemia [6]. Low high-density lipoprotein cholesterol levels (HDL-C) are defined as less than 40 mmol/L in females and less than 50 mmol/L in males. Additionally, prediabetes is characterized by any of the following: impaired fasting glucose, impaired glucose tolerance, or glycated hemoglobin levels ranging from 5.7% to 6.4%. Insulin resistance is indicated by a homeostasis model assessment (HOMA) score of 2.5 or higher, while subclinical inflammation is evidenced by plasma high-sensitivity C-reactive protein levels exceeding 2 mg/L. All these factors are associated with MASLD [5].

Moreover, MASLD is associated not only with a heightened risk of hepatocellular carcinoma but also with cardiovascular diseases and complications related to type 2 diabetes, such as nephropathy and neuropathy. Thus, it is essential to delineate the pathways that contribute to the pathophysiology of MASLD for effective management of the condition and its associated complications [6]. The pathogenesis of MASLD appears to involve a cyclical process of steatosis, lipotoxicity, and inflammation that leads to complex changes in the liver’s histopathological and biochemical characteristics [7]. Furthermore, these pathogenic mechanisms lead to the redistribution of adipose tissue from the lower body to the upper body and from subcutaneous to visceral fat deposits, increasing the amount of lipids stored ectopically in the liver [8].

During the development of MASLD, hepatic inflammatory cytokines, reactive oxygen species, and products of lipid peroxidation are all induced. Numerous cytokines are released as a consequence, which play essential roles in inflammation, fibrosis, and cell death [5]. Damaged hepatocytes trigger the Kupffer cells, the indigenous hepatic macrophages, which release proinflammatory cytokines such as tumor necrosis factor (TNF-alpha), interleukin-1beta (IL-1b), and interleukin-6 [6]. High-sensitivity C-reactive protein (hs-CRP) is another inflammatory marker associated with liver inflammation [7]. HsCRP levels were significantly higher in individuals with MASLD and strongly correlated with the disease severity of MASLD [8].

Trimetazidine (TMZ) is an inhibitor of free fatty acid (FFA) oxidation that redirects muscle and cardiac metabolism to sugar use, leading to a greater generation of high-energy phosphates [9]. Recently, it has gained attention for its potential therapeutic applications beyond its primary uses. It works in various ways, including enhancing vasodilation, decreasing oxidative stress and inflammation, and regulating apoptosis and angiogenesis. Additionally, it may help to reduce atherosclerosis and lower blood sugar levels [10].

Trimetazidine is effective mainly through inhibiting fatty acid β-oxidation, encouraging the oxidation of glucose, and reducing inflammation (IL-6 and TNF-α) [11]. Also, it shifts muscle metabolism towards glucose utilization, increasing the synthesis of high-energy phosphate [12]. Furthermore, a recent preclinical study showed that trimetazidine pre-treatment reduces liver damage, promotes liver regeneration, and increases survival rates in an experimental model of partial hepatectomy with hepatic circulation obstruction [13].

TMZ significantly decreased hepatocyte bullous steatosis and protected against hepatic fibrosis, and it has been recognized as a metabolic medication with hypolipidemic properties [14]. This effect has been discussed in earlier research [14,15,16,17,18].

The purpose of this study was to evaluate the potential benefits of trimetazidine for individuals with MASLD who exhibited liver steatosis, fibrosis, inflammation, and elevated liver enzymes.

## 2. Results

**Figure 1:**
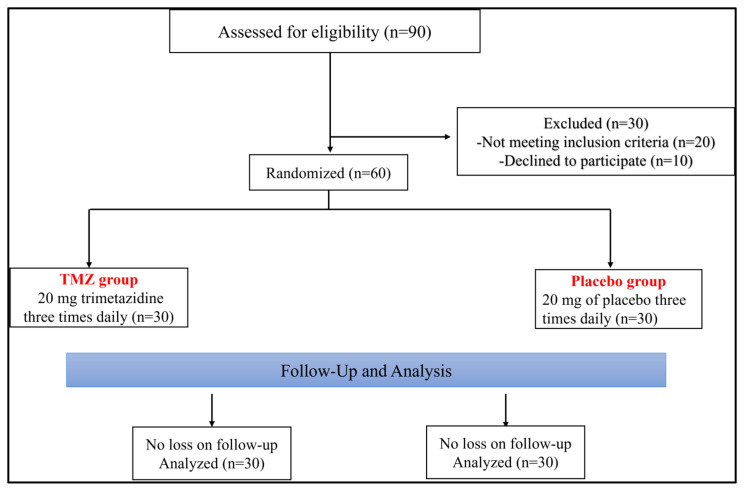
Flowchart of the study.

Of the 90 patients screened, 60 were allocated to the trial, while 30 did not meet the inclusion criteria. They were divided into two groups, each with thirty patients. The patient flowchart is presented in Figure 1.

### 2.1. Baseline Characteristics and Homogeneity Between Groups

The baseline characteristics were notably consistent between the two study groups, with no statistically significant differences identified in any of the evaluated parameters (see Table 1). The non-significant comparisons at baseline encompassed age, body weight, sex, height, body mass index (BMI), and waist circumference (all *p* > 0.05). Also, there was no significant difference between the two groups in terms of comorbidity, including hypertension and diabetes. In terms of metabolic parameters, there were no significant differences in fasting blood sugar (FBS), homeostatic model assessment for insulin resistance (HOMA-IR), total cholesterol (TC), triglycerides (TG), high-density lipoprotein cholesterol (HDL), and low-density lipoprotein cholesterol (LDL) (all *p* > 0.05). Liver biochemical markers demonstrated comparability, with gamma-glutamyl transferase (GGT) and aspartate aminotransferase (AST) showing borderline non-significant differences (both *p* > 0.05), while alanine aminotransferase (ALT) revealed a significant difference (*p* = 0.004).

Additionally, the baseline non-invasive fibrosis scores showed no significant differences, as indicated by the AST-to-platelets ratio index (APRI score), Fibrosis-4 index (FIB-4), non-alcoholic fatty liver disease score (NAFLD score), atherosclerotic cardiovascular disease score (ASCVD), and transient elastography (TE), all with *p*-values greater than 0.05. Regarding steatosis measurements via the controlled attenuation parameter (CAP score), baseline values were also similar (*p* > 0.05). Conversely, the fibrosis to AST score (FAST score) indicated minor baseline differences (*p* = 0.020). However, inflammatory markers displayed non-significant differences in Interleukin-6 (IL-6) and highly sensitive C-reactive protein (Hs-CRP) levels. However, tumor necrosis factor alpha (TNF-α) demonstrated a significant difference (*p* < 0.001).

### 2.2. Treatment Outcomes After 24 Weeks

After 24 weeks of treatment (Table 2), regarding inflammation, TNF-alpha showed a significant reduction (*p* = 0.001) between the two groups.

**Figure 2:**
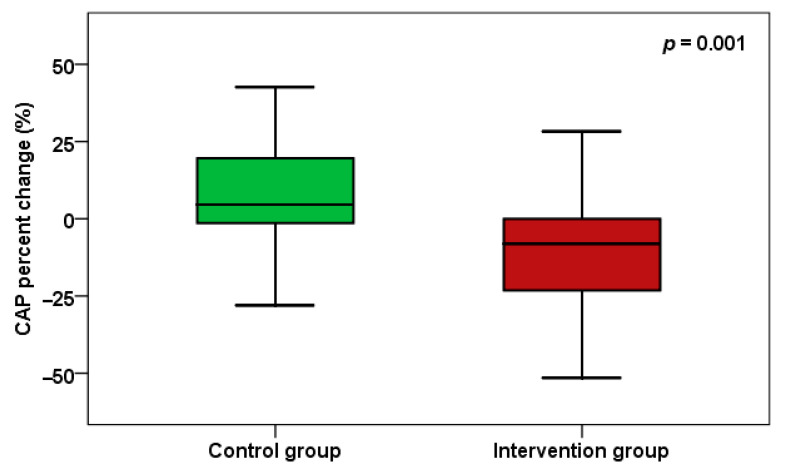
CAP score percent change between the two groups.

**Figure 3:**
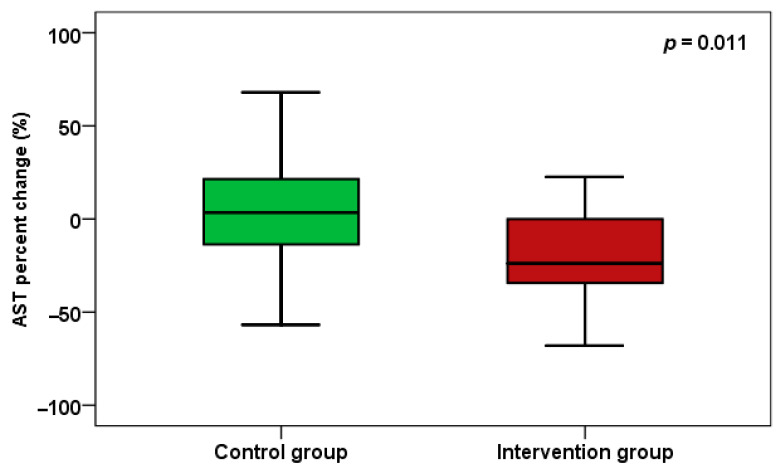
AST percent change between the two groups.

Regarding liver fibrosis and steatosis, CAP shows improvement, with a significant difference between groups (*p* < 0.001) and a percent change at *p* = 0.001 (Figure 2). Regarding aminotransferases, AST in the trimetazidine group showed a significant decrease (*p* < 0.001), and AST also demonstrated a significant difference between groups (*p* = 0.032), with a percent change of *p* = 0.011 (Figure 3).

**Figure 4:**
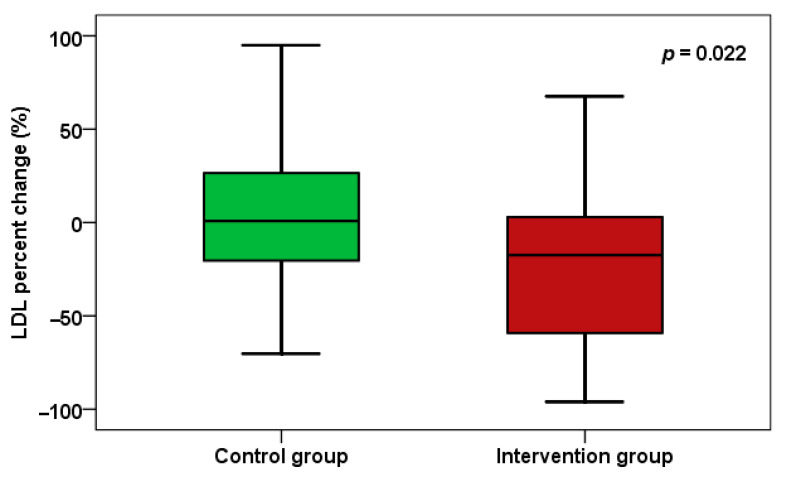
LDL cholesterol percent change between the two groups.

**Figure 5:**
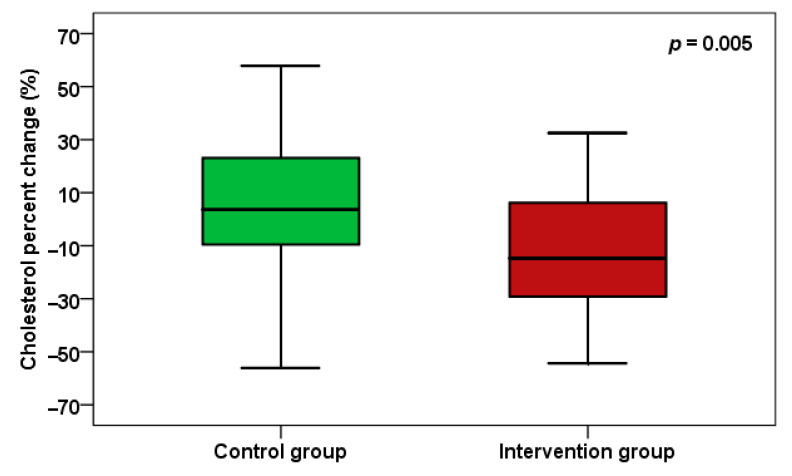
Total cholesterol percent change between the two groups.

Regarding the lipid profile and metabolic parameters after 24 weeks, LDL cholesterol levels decreased significantly (*p* = 0.004) between the two groups, with a percent change of *p* = 0.022 (Figure 4). There was also a significant reduction in total cholesterol (*p* = 0.004 between groups), with a percent change of *p* = 0.005 (Figure 5). Additionally, there were no significant changes in HDL cholesterol and other metabolic measures. Additionally, there were no significant alterations in HOMA-IR and fasting blood glucose between the two groups. Notably, there were no significant side effects observed over the 24 weeks in either group.

## 3. Discussion

Trimetazidine (TMZ) has emerged as a potential therapeutic candidate for MASLD, with previous studies demonstrating its efficacy in animal models [12,13,14,15]. In this 24-week randomized, double-blind, placebo-controlled trial, TMZ produced significant improvements in hepatic steatosis and fibrosis in patients with MASLD. These effects were demonstrated using multiple non-invasive measures, including Fibro-Scan and CAP scores, and were accompanied by reductions in serum aminotransferase levels. TMZ also improved lipid parameters, with significant decreases in total cholesterol and LDL cholesterol compared with placebo, whereas no significant effects were observed for HDL cholesterol or triglycerides.

The lipid-lowering profile observed in this trial aligns with previous evidence showing reductions in LDL and total cholesterol without consistent changes in HDL or triglycerides over similar treatment durations [19,20,21,22], as HDL and triglycerides levels may require longer treatment durations to shift meaningfully, as suggested by longer-term studies, or may benefit from combination therapy with statins, which has been shown to enhance lipid improvements [19,22].

The anti-steatotic and anti-fibrotic effects observed in our study are also in agreement with prior research. Consistent with these findings, several preclinical studies have demonstrated the hepatoprotective effects of TMZ in models of liver injury. For example, pretreatment with TMZ reduced fibrosis, attenuated liver enzyme elevations, and improved survival in experimental hepatic injury models [12,13,14,15,23]. Similarly, in a murine model of pancreatitis, TMZ administration significantly reduced serum AST, further supporting its role in limiting hepatocellular damage [24].

Moreover, previous studies have suggested that TMZ may help protect against hepatic injury, showing improvements in liver function and steatosis, with additional potential benefits in surgical models such as enhanced recovery following hepatic flow occlusion for partial hepatectomy [12,14]. These data provide additional biological plausibility to our clinical observations.

Beyond its impact on steatosis and fibrosis, trimetazidine also exerted a notable anti-inflammatory effect, resulting in a significant reduction in TNF-α levels compared with placebo, which is consistent with earlier clinical reports demonstrating anti-inflammatory effects in metabolic liver disease [19,23,24,25,26]. In contrast, IL-6 and hs-CRP levels did not change significantly over the 24-week intervention, differing from some previous findings in other clinical populations [18,19]. These discrepancies may reflect differences in study duration, population characteristics, or disease severity. An interesting observation was the improvement in inflammatory markers in some participants in the placebo group, likely related to lifestyle modifications undertaken during the study. Such changes, including improved diet and increased physical activity, can reduce systemic inflammation even before biochemical or imaging evidence of hepatic recovery becomes apparent [27,28].

When compared with other metabolic modulators for MASLD, the magnitude and pattern of TMZ’s effects suggest a potentially complementary role. For example, glucagon-like peptide-1 receptor agonists (GLP-1 RAs) such as semaglutide improve hepatic outcomes primarily through weight loss and glycemic control, whereas sodium-glucose cotransporter-2 (SGLT2) inhibitors improve steatosis, fibrosis, and liver enzymes primarily via weight reduction, visceral fat loss, and improved glycemia [29,30]. Pioglitazone has demonstrated histological benefits in MASLD, but may be associated with weight gain and fluid retention [31]. In contrast, TMZ appears to act without substantial weight change, offering an alternative or adjunctive option for patients who may not tolerate these other therapies or in whom additional metabolic optimization is needed.

This study had several limitations. First, the sample size was relatively small, which may have limited the statistical power for some secondary endpoints. Second, recruiting all participants from a single health setting could limit the study’s generalizability. Third, liver biopsy or MRI, considered the gold standard for MASLD diagnosis and staging, could not be used for ethical reasons. Finally, despite randomization, there were baseline imbalances in ALT levels, FAST scores, and TNF-α concentrations between groups, which were addressed using ANCOVA but could still introduce residual confounding.

Nevertheless, the trial’s strengths include its double-blind, placebo-controlled design, standardized control of concurrent medications, and exclusion of agents known to improve MASLD outcomes (such as GLP-1 RAs, SGLT2 inhibitors, and statins) in order to better isolate the effects of TMZ. Taken together, our findings suggest that TMZ may represent a promising adjunctive therapy for MASLD, warranting further investigation in larger, multicenter trials with longer follow-up and in combination with other evidence-based treatments.

## 4. Materials and Methods

### 4.1. Study Design and Ethical Considerations

A double-blind, placebo-controlled trial was conducted at Beni-Suef University Hospital from December 2023 to November 2024. The research protocol adhered to the principles of the Declaration of Helsinki and received approval from the hospital’s Ethics Committee. The Clinical Trials Registry registration number for this trial is NCT06140953. Prior to enrollment, written informed consent was obtained from each participant. As illustrated in Figure 1, the study was a double-masked, placebo-controlled experiment lasting 24 weeks, with participants randomly assigned using a computer-generated allocation sequence. Allocation concealment was ensured using sealed opaque envelopes. Participants were randomized into two groups (1:1) to receive either trimetazidine 20 mg orally three times a day, along with lifestyle adjustments, or a placebo combined with lifestyle modifications. Both the active medication and the placebo tablet were identical in form, color, and packaging. Throughout the study, the trimetazidine group received a total of 60 mg daily.

### 4.2. Patient Selection

Patients were enrolled in the study if they met the diagnostic criteria for metabolic-associated steatotic liver disease (MASLD), including specific metabolic factors such as a BMI of 25 kg/m^2^ or higher, the presence of diabetes or dysglycemia, blood pressure readings of 130/85 mmHg or greater, triglyceride levels of 150 mg/dL or more, or reduced levels of HDL-cholesterol. Additionally, participants were required to have imaging-confirmed hepatic steatosis, as determined by abdominal ultrasound and CAP measurements.

Those with alternative causes of steatosis, including drug-induced liver damage, hepatitis C, Wilson’s disease, or alcohol consumption exceeding 50 g per day for females and 60 g per day for males, were excluded from the study. Further exclusion criteria included the presence of hepatocellular carcinoma, decompensated cirrhosis, portal hypertension, pregnancy, individuals under the age of 18, and contraindications for trimetazidine—such as Parkinson’s disease, parkinsonian symptoms, tremors, and severe renal impairment. Additionally, patients who were on statins, (SGLT2), or (GLP-1) were also excluded, as these medications could impact liver steatosis and glycemic parameters, potentially confounding the efficacy of trimetazidine. However, other standardized medications (such as antihypertensives, anti-diabetics, and anti-hyperlipidemic) that did not include those mentioned above, excluding MASLD-specific agents, were permitted.

### 4.3. Clinical Assessment

The clinical evaluation consisted of a detailed medical history, which included information on current medications, comorbidities, lifestyle factors, and demographics. During the physical examination, measurements were taken of waist circumference (normal values: ≤102 cm for men and ≤88 cm for women), BMI, and blood pressure (with hypertension defined as ≥130/80 mmHg or current antihypertensive treatment).

### 4.4. Laboratory Investigations

The assessments conducted included a complete blood count (CBC), which was performed using the Sysmex XP-300 automated hematology analyzer (Sysmex, Kobe, Japan), a metabolic panel, and lipid measurements, such as LDL cholesterol, HDL cholesterol, TG, total cholesterol, ALT, AST and GGT, all analyzed using the photometric method with Beckman kit on the Beckman Coulter AU 480 analyzer (Beckman Coulter Ireland Inc., O’Callaghans Mills, Ireland). Additionally, hemoglobin A1c (HbA1c) was determined by the turbidimetric method on the same analyzer. Fasting insulin and glucose levels were measured to calculate the HOMA-IR, which is computed by multiplying fasting insulin, quantified by chemiluminescence immunoassay (CLIA) (μU/mL), by fasting blood sugar measured using the Beckman Coulter AU 480 (mmol/dL) and then dividing by 22.5. Non-invasive fibrosis assessment employed various scoring systems: APRI is calculated as [(AST/ULN)/platelet count (10^9^/L)] × 100, with a threshold of 0.7 demonstrating 77% sensitivity and 72% specificity [32]; FIB-4 index as [Age (years) × AST (U/L)]/[platelet count (10^9^/L) × √ALT (U/L)] (<1.45 low, 1.45–3.25 intermediate, >3.25 high fibrosis probability) [33], NAFLD fibrosis score [34] (<−1.5 low, −1.5 to 0.67 intermediate, ≥0.67 high probability); ASCVD 10-year risk score (<5% low, 5–7.5% borderline, >7.5–<20% intermediate, >20% high risk) [35], and FAST score (≥0.35 high, ≤0.25 low, 0.25–0.35 intermediate probability) [36]. Serum (IL-6) was measured by ELISA using human IL-6 ELISA kit (CAT.NO.:[SL1001Hu] (SunLog Biotech Co., Ltd., Hangzhou, China) on Stat Fax-2100 reader and Hydro flex washer (Tecan Austria, GmbH, Grödig, Austria); TNF-alpha was measured using TNF-α ELISA kit (CAT.NO.: [SL1761Hu]) (SunLog Biotech Co., Ltd., Hangzhou, China) on Stat Fax-2100 reader and Hydro flex washer (Tecan Austria, GmbH, Grödig, Austria); and hs-CRP was measured using CRP-hs kit (Roch Diagnostic GmbH, Mannheim, Germany) on Cobas pro analyzer (Roch Diagnostic GmbH, Mannheim, Germany), based on the chemiluminescence immunoassay (CLIA) method.

### 4.5. Imaging Studies

Abdominal ultrasonography was employed to assess liver echogenicity, hepatomegaly, portal hypertension signs, and focal lesions [37]. The Fibro-Scan Touch 502, equipped with an XL probe (Echosens, Paris, France), was used to assess liver stiffness.

Moreover, CAP values indicated <237 dB/m (no steatosis), 237.0–259.0 dB/m (mild), 259.0–291.0 dB/m (moderate), and 291.0–400.0 dB/m (severe) [38]. LSM values [39] were designated as follows: 7.9 kPa for F0-F1, 7.9–< 8.8 kPa for F2, 8.8–<11.7 kPa for F3, and 11.7 kPa for F4 α.

### 4.6. Follow-Up Protocol

After six months of therapy, both groups underwent re-evaluation, including clinical evaluation, laboratory analyses, transient elastography, and recalibration of non-invasive fibrosis indices.

### 4.7. Sample Size Calculation

As we have two treatment groups, we performed a two-sample *t*-test to assess effect size and the required number of patients per group from a previous study based on the (hs-CRP) measurement [40]. Given that α = 0.01 and β = 0.01 (implying the power of 0.95), the required sample size per group is 24. Considering a 25% dropout, each group should include 30 patients. In addition, Cohen’s 1.269183.

### 4.8. Statistical Methods

IBM SPSS^®^ Statistics version 23 (IBM^®^ Corp., Armonk, NY, USA) was used for statistical analysis. Numerical data were represented using the mean and standard deviation or, when appropriate, the median and range. Frequencies and percentages were employed to convey qualitative data.

Data were tested for normality using the Kolmogorov–Smirnov test and the Shapiro–Wilk test.

Pearson’s Chi-square test was used to examine the relationship between qualitative variables (gender).

To compare quantitative variables between the two groups, either the Student’s *t*-test for normally distributed data, including hemoglobin, platelets, white blood cells, and albumin, weight, height, waist, BMI, age, or the Mann–Whitney test (a non-parametric *t*-test) was applied for numerically distributed data, including LDL, HDL, TG, total cholesterol, ALT, AST, GGT, HOMA-IR, FBG, CAP, FAST, NAFLD, APRI, ASCVD, FIB4 scores, IL-6, TNF-alpha, and hs-CRP.

The Wilcoxon Signed Ranks test (a non-parametric paired *t*-test) was employed to compare LDL, HDL, TG, total cholesterol, ALT, AST, GGT, HOMA-IR, FBG, CAP, FAST, NAFLD, APRI, ASCVD, FIB4 scores, IL-6, TNF-alpha, and hs-CRP within each group.

A paired *t*-test was used to compare the levels of hemoglobin, platelets, white blood cells, and albumin in each group. These tests were implemented to assess the consistency of two consecutive measurements of numerical variables.

Due to multiple comparisons, the *p*-value was corrected using the Bonferroni method.

All statistical tests were two-tailed, with a *p*-value of less than 0.05 considered statistically significant. Baseline comparisons revealed notable differences in several key variables, including TNF-α, the FAST score, and ALT, despite random allocation. To address these imbalances and ensure accurate estimation of treatment effects, analysis of covariance (ANCOVA) was applied to these outcomes.

## 5. Conclusions

This randomized controlled trial demonstrates that a 24-week regimen of trimetazidine, combined with lifestyle modifications, significantly improved hepatic steatosis, inflammatory markers, and lipid profiles in patients with MASLD, compared to those receiving a placebo. These promising findings underscore the need for larger multicenter trials with extended follow-up to confirm long-term effectiveness, identify optimal dosing, and clarify the mechanisms underlying the therapeutic effects of trimetazidine in MASLD.

## Figures and Tables

**Table 1:** Comparison of baseline parameters between the two groups.

Variable	Control (*n* = 30)	Trimetazidine (*n* = 30)	*p* Value
Age (years) *^a^*	47.3 ± 7.6	49.8 ± 7.5	0.206
Sex #			
Male	12 (40%)	14 (46.7%)	0.602
Female	18 (60%)	16 (53.3%)	
Weight (kg) *^a^*	96 ± 17.8	93.7 ± 14.4	0.595
height(cm) *^a^*	160.8 ± 8.8	159 ± 12.3	0.517
BMI (kg/m^2^) *^a^*	37.16 ± 6.53	37.33 ± 6.71	0.922
waist(cm) *^a^*	115.6 ± 12.9	115.5 ± 11.4	0.975
HB(g/dL) *^a^*	13.5 ± 1.8	13.4 ± 1.3	0.821
Platelets(×10^9^/L) *^a^*	276.2 ± 53.8	281.2 ± 68.4	0.754
WBCS (wbcs/mcl) *^a^*	6.66 ± 1.79	6.67 ± 2.40	0.990
Albumin(g/dL) *^a^*	3.9 ± 0.4	4.1 ± 0.4	0.296
Total Chol(mg/dL) *^b^*	198 (119–276)	191 (113–376)	0.371
TG (mg/dL) *^b^*	163.5(87–481)	180.5 (96–403)	0.706
HDL (mg/dL) *^b^*	50.5 (31–63)	44.5 (30–80)	0.553
LDL (mg/dL) *^b^*	102.90 (25–181.8)	103.70 (18.20–378)	0.813
GGT (mg/dL) *^b^*	40(16–121)	38 (14–445)	0.824
AST (IU/L)^*b*^	28 (20–66)	35.5 (15–75)	0.060
ALT (IU/L) *^b^*	25.5 (17–64)	31.5 (19–73)	0.004 **
FBG (mg/dL) *^b^*	103 (82–240)	112 (67–372)	0.214
HOMA-IR *^b^*	1.95 (0.96–4)	1.95 (0.90–5.80)	0.491
CAP (dB/m) *^b^*	328.5 (100–381)	352.5 (207–400)	0.184
FAST *^b^*	0.25 (0.08–0.74)	0.49 (0.06–0.74)	0.020 *
APRI *^b^*	0.2 (0.1–0.9)	0.3 (0.2–1.1)	0.083
FIB4 *^b^*	0.96 (0.49–2.77)	1.05 (0.51–3.96)	0.344
NAFLD score *^b^*	−1.19 (−3.42–1.79)	−1.36 (−3.86–2.15)	0.673
ASCVD score *^b^*	2 (0.40–35.30)	2.70 (0.30–22.05)	0.322
Fibrosis *^b^*	5.8 (3.1–15.5)	6.9 (2.9–21.1)	0.399
IL6 (pg/L) *^b^*	17.3 (0–28.5)	17.6 (0–68.3)	0.953
TNF alpha (pg/mL) *^b^*	20.4 (0–68)	67.9 (17.3–109.6)	<0.001 **
HsCRP (mg/dL) *^b^*	11.3 (2.5–44.9)	10.7 (1.2–43.5)	0.438

*^a^* represents data as mean ± SD. *^b^* represents data as median (IQR). # represents data as a number (percent). Abbreviations: BMI: body mass index, HB: hemoglobin, total Chol: total cholesterol, TG: triglycerides, HDL: high-density lipoprotein, LDL: low-density lipoprotein, GGT: gamma-glutamyl transferase, AST: aspartate transferase, ALT: alanine transaminase, FBG: fasting blood glucose, HOMA-IR: homeostasis model assessment of insulin resistance, CAP: controlled attenuated parameter, FAST: Fibro-Scan-AST, APRI: AST, platelet ratio index, FIB4: fibrosis-4 index, NAFLD: non-alcoholic fatty liver disease, ASCVD: atherosclerotic cardiovascular disease, IL6: interleukin 6, TNF alpha: tumor necrosis factor, HsCRP: highly sensitive c-reactive protein. * Significant difference at *p*-value < 0.05. ** Significant difference at *p*-value < 0.01.

**Table 2:** Comparison of parameters at baseline and after 6 months within and between the placebo group and the trimetazidine group.

Parameter	Placebo Group	*p*-Value	TMZ Group	*p*-Value	*p*-Value Between Two Groups After 6 Months
Total Chol (mg/dL) *^b^*					
Baseline	198 (119–276)	0.271	191 (113–376)	0.020 *	0.004 **
After 6 months	208.5 (104–286)		165 (99–261)		
TG (mg/dL) *^b^*					
Baseline	163.5 (87–481)	0.742	180.5 (96–403)	0.176	0.132
After 6 months	196.5 (90–403)		152 (66–797)		
HDL (mg/dL) *^b^*					
Baseline	50.5 (31–63)	0.657	44.5 (30–80)	0.726	1000
After 6 months	47 (35–85)		48.5 (26–70)		
LDL (mg/dL) *^b^*					
Baseline	102.9 (25–181.8)	0.192	103.7 (18.2–378)	0.020 *	0.004 **
After 6 months	114.4 (41.2–200.6)		86.6 (3.2–152.2)		
AST (IU/L) *^b^*					
Baseline	28 (20–66)	0.206	35.5 (15–75)	<0.001 **	0.032 **
After 6 months	34 (17–63)		27 (18–57)		
ALT (IU/L) *^b^*					
Baseline	25.5 (17–64)	0.194	31.5 (19–73)	0.004 **	0.08 *t*
After 6 months	31.5 (19–64)		26.5 (16–85)		
GGT (mg/dL) *^b^*					
Baseline	40 (16–121)	0.666	38 (14–445)	0.084	0.329
After 6 months	39.5 (14–127)		35.5 (18–228)		
FBG (mg/dL) *^b^*					
Baseline	103 (82–240)	0.558	112 (67–372)	0.072	0.662
After 6 months	102 (78–334)		106 (60–211)		
HOMA-IR *^b^*					
Baseline	1.95 (0.96–4)	0.593	1.95 (0.90–5.8)	0.758	0.420
After 6 months	1.85 (0.86–6.40)		2 (1.03–5.10)		
Albumin (g/L) *^a^*					
Baseline	3.9 ± 0.4	0.187	4.1 ± 0.39	0.500	0.299
After 6 months	4 ± 0.3		4.07 ± 0.28		
HB (g/L) *^a^*					
Baseline	13.5 ± 1.8	0.656	13.38 ± 1.33	0.389	0.743
After 6 months	13.3 ± 1.5		13.13 ± 1.38		
Platelets (×10^9^/L) *^a^*					
Baseline	276.2 ± 53.8	0.395	281.23 ± 68.36	0.195	0.912
After 6 months	265 ± 63.9		263.3 ± 51.32		
WBCS (wbcs/mcl) *^a^*					
Baseline	6.7 ± 1.8	0.352	6.67 ± 2.40	0.209	0.916
After 6 months	7.1 ± 2.2		7.20 ± 2.81		
CAP (dB/m) *^b^*					
Baseline	328.5 (100–381)	0.072	352.5 (207–400)	0.008 **	<0.001 **
After 6 months	348 (270–400)		302 (124–400)		
FAST *^b^*					
Baseline	0.25 (0.08–0.74)	0.292	0.49 (0.06–0.74)	<0.001 **	0.0569 *t*
After 6 months	0.38 (0.06–0.71)		0.25 (0.04–0.77)		
APRI *^b^*					
Baseline	0.2 (0.1–0.9)	0.268	0.3 (0.2–1.1)	0.204	0.065
After 6 months	0.3 (0.2–0.7)		0.3 (0.1–0.6)		
FIB4 *^b^*					
Baseline	0.96 (0.49–2.77)	0.524	1.05 (0.51–3.96)	0.120	0.261
After 6 months	1.07 (0.47–2.14)		0.84 (0.43–3.20)		
NAFLD Score *^b^*					
Baseline	−1.19 (−3.42–1.79)	0.558	−1.36 (−3.86–2.15)	0.144	0.595
After 6 months	−1 (−3.54–1.52)		−1.04 (−2.59–2.15)		
ASCVD Score *^b^*					
Baseline	2 (0.4–35.30)	0.894	2.70 (0.30–22.05)	0.336	0.762
After 6 months	1.7 (0.7–20.5)		2.2 (0.3–18.9)		
Fibrosis *^b^*					
Baseline	5.8 (3.1–15.5)	0.746	6.9 (2.9–21.1)	0.991	0.284
After 6 months	5.1 (2.9–28)		7.1 (2.1–18.8)		
IL6 (pg./L) *^b^*					
Baseline	17.3 (0–28.5)	0.004 **	17.6 (0–68.3)	0.004 **	0.784
After 6 months	12.7 (0–21.6)		12.8 (0–26.1)		
TNF alpha(pg./mL) *^b^*					
Baseline	20.4 (0–68)	0.004 **	67.9 (17.3–109.6)	0.096	0.001 ** *t*
After 6 months	17.6 (0–52.5)		42.7 (8.3–103)		
HsCRP (mg/dL) *^b^*					
Baseline	11.3 (2.5–44.9)	<0.001 **	10.7 (1.2–43.5)	<0.001 **	0.336
After 6 months	8.4 (1–35.1)		5.7 (0.5–40.2)		

*^a^* represents data as mean ± SD. *^b^* represents data as median (IQR). *t* by ANCOVA. Key findings from this table are also presented graphically in the Supplementary Materials. Abbreviations: HB: hemoglobin, total Chol: total cholesterol, TG: triglycerides, HDL: high-density lipoprotein, LDL: low-density lipoprotein, GGT: gamma-glutamyl transferase, AST: aspartate transferase, ALT: alanine transaminase, FBG: fasting blood glucose, HOMA-IR: homeostasis model assessment of insulin resistance, CAP: controlled attenuated parameter, FAST: Fibro-Scan, AST, APRI: AST, platelet ratio index, FIB4: fibrosis-4 index, NAFLD: non-alcoholic fatty liver disease, ASCVD: atherosclerotic cardiovascular disease, IL6: interleukin 6, TNF alpha: tumor necrosis factor, HsCRP: highly sensitive c-reactive protein. * Significant difference at *p*-value < 0.05. ** Significant difference at *p*-value < 0.01.

## Data Availability

The authors confirm that the data supporting the findings of this study are available within the article. Raw data supporting the findings of this study are available from the first author upon reasonable request.

## Supplementary Materials

The following supporting information can be downloaded at: https://www.mdpi.com/article/10.3390/ph18091279/s1, Figure S1: CAP score levels at baseline and after 6 months in the control and the intervention group. Figure S2: AST levels at baseline and after 6 months in the control and the intervention group. Figure S3: LDL levels at baseline and after 6 months in the control and the intervention group. Figure S4: Cholesterol levels at baseline and after 6 months in the control and the intervention group.

## Abbreviations

MASLD: metabolic dysfunction-associated steatotic liver disease, MASH: metabolic associated steatohepatitis, CRP: c-reactive protein, IL: interleukin, TNF alpha: tumor necrosis factor alpha, FAST: FibroScane-AST, CAP: controlled attenuated parameter, TE: transient elastography, AST: aspartate aminotransferase, ALT: alanine aminotransferase, LDL: low-density lipoprotein, FBS: fasting blood sugar, HBA1C: hemoglobin A1C, CBC: complete blood count, TE: trans elastography, TMZ: trimetazidine, TC: total cholesterol, TG: triglycerides, HDL: high-density lipoprotein, T2DM: type 2 diabetes mellites, HOMA-IR: homeostasis model assessment, FFA: free fatty acids, BMI: body mass index, GLP1: glucagon-like peptide1, SGLT2: sodium glucose transporter 2, APRI: aspartate aminotransferase to platelet ratio, FIB4: fibrosis 4 index, NAFLD: non-alcoholic fatty liver, LSM: liver stiffness measurements, GGT: gamma-glutamyl transferase, ASCVD: atherosclerotic cardiovascular disease score, ATP: adenosine triphosphate, NOX: nitric oxide, MRI: magnetic resonance imaging, AMPK: AMP activated protein kinase, CHREBP: carbohydrate response element binding protein, NOT: NADPH oxidase-mediated rotenone activity.
