# Supplementary material for: Evaluation of the Potential Benefits of Trimetazidine in Metabolic Dysfunction-Associated Steatotic Liver Disease: A Randomized Controlled Trial
Source: Pharmaceuticals (Basel). 2025 Aug 27;18(9):1279. doi: 10.3390/ph18091279 (PMC12472768; doi:10.3390/ph18091279)
Supplement: Supplementary file 1 [file pharmaceuticals-18-01279-s001.zip › pharmaceuticals-3785814-supplementary.pdf]

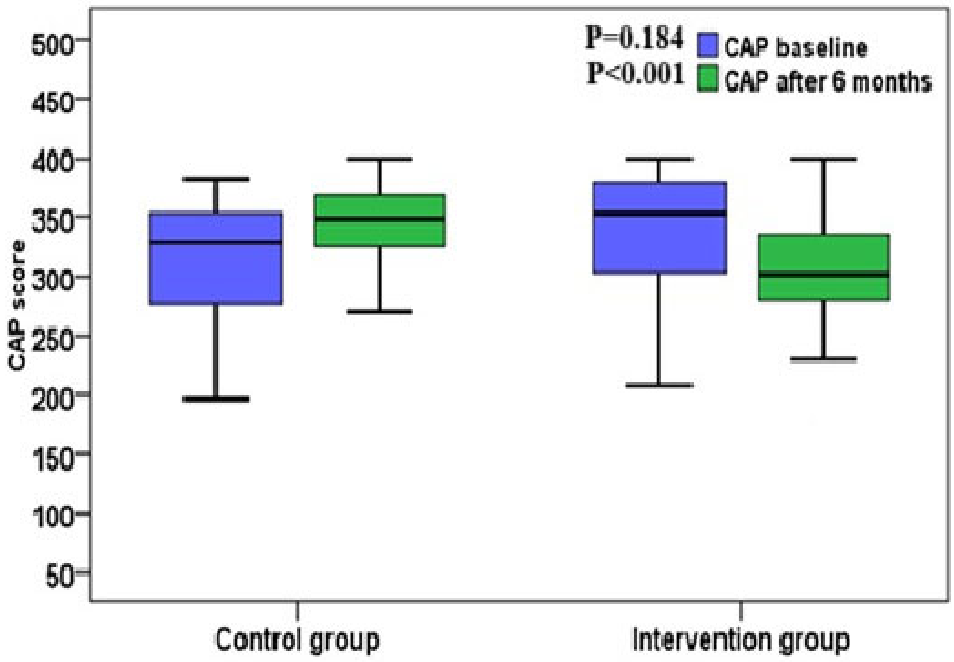


**Figure S1:** CAP score levels at baseline and after 6 months in the control and the intervention group

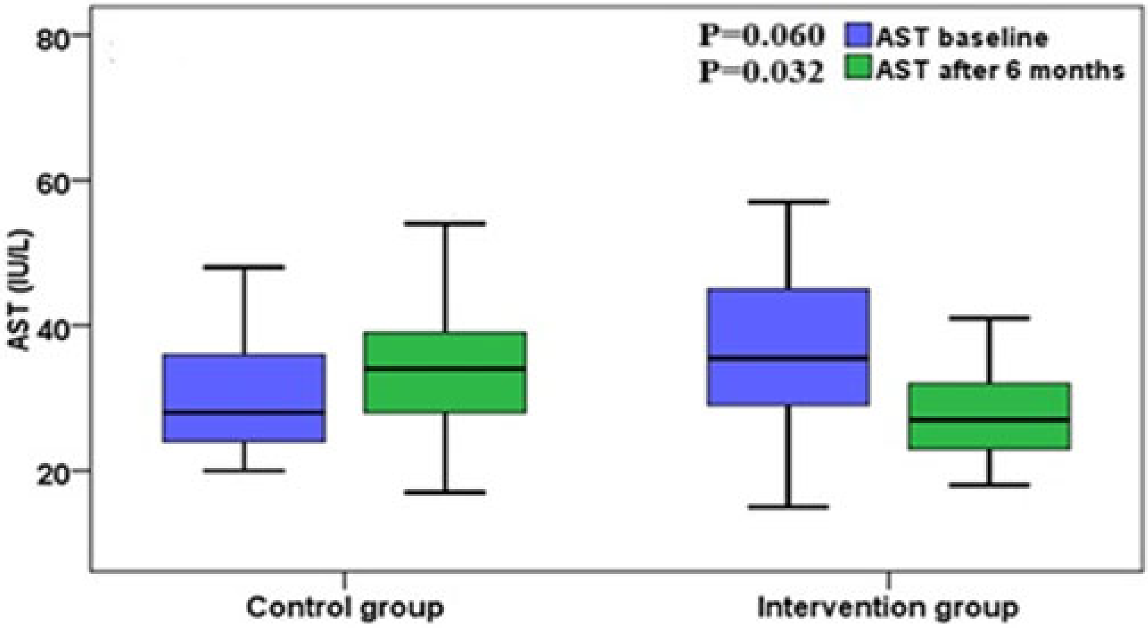


**Figure S2:** AST levels at baseline and after 6 months in the control and the intervention group

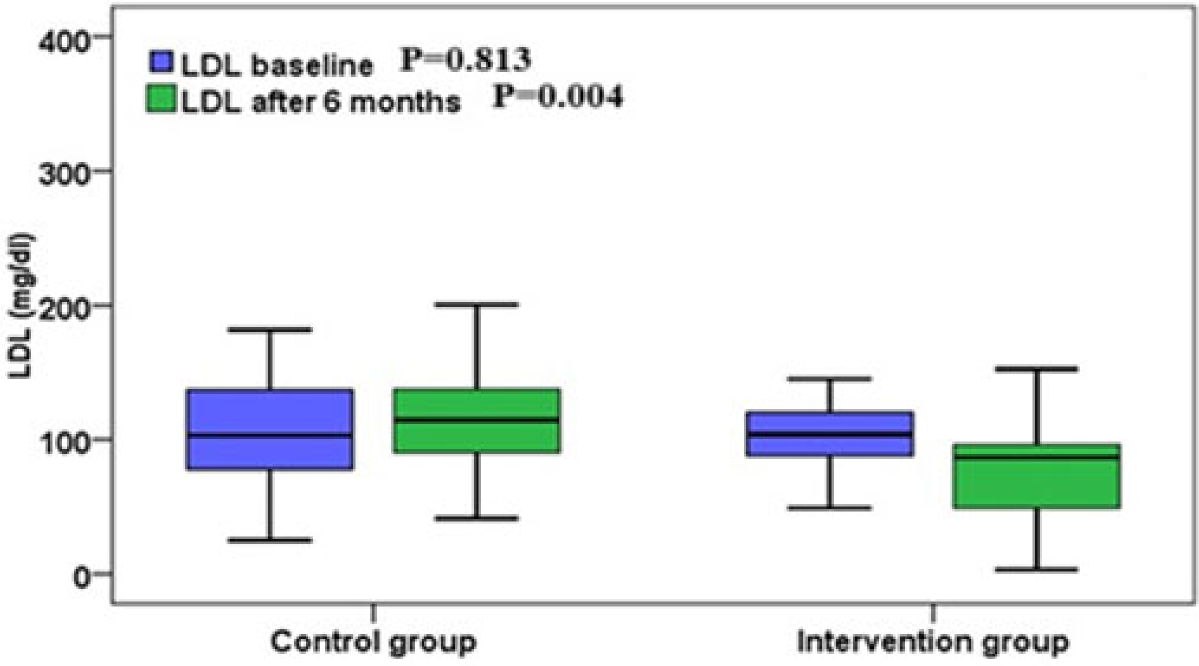


**Figure S3:** LDL levels at baseline and after 6 months in the control and the intervention group

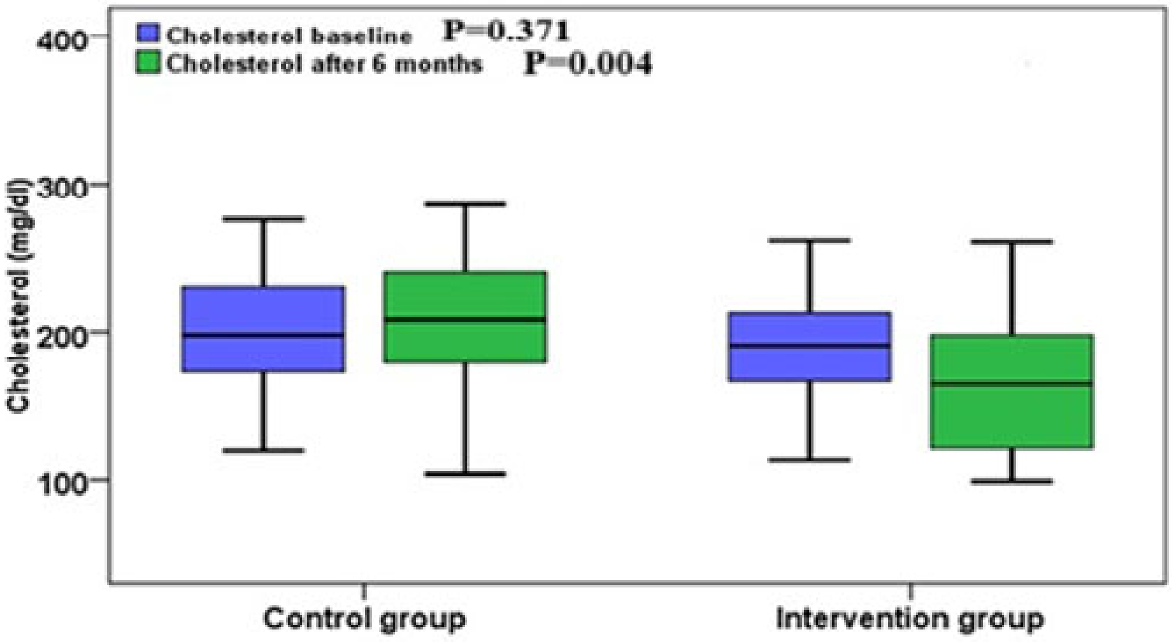


**Figure S4:** Cholesterol levels at baseline and after 6 months in the control and the intervention group
